# Cell wall-related genes and lignin accumulation contribute to the root resistance in different maize (*Zea mays* L.) genotypes to *Fusarium verticillioides* (Sacc.) Nirenberg infection

**DOI:** 10.3389/fpls.2023.1195794

**Published:** 2023-06-27

**Authors:** Francisco Roberto Quiroz-Figueroa, Abraham Cruz-Mendívil, Enrique Ibarra-Laclette, Luz María García-Pérez, Rosa Luz Gómez-Peraza, Greta Hanako-Rosas, Eliel Ruíz-May, Apolinar Santamaría-Miranda, Rupesh Kumar Singh, Gerardo Campos-Rivero, Elpidio García-Ramírez, José Alberto Narváez-Zapata

**Affiliations:** ^1^ Instituto Politécnico Nacional, Centro Interdisciplinario de Investigación para el Desarrollo Integral Regional (CIIDIR)—Unidad Sinaloa, Guasave, Mexico; ^2^ Consejo Nacional de Ciencia y Tecnología (CONACYT)-Instituto Politécnico Nacional, (CIIDIR) Unidad Sinaloa, Guasave, Mexico; ^3^ Red de Estudios Moleculares Avanzados, Instituto de Ecología A. C., Cluster BioMimic®, Xalapa, Mexico; ^4^ Centre of Molecular and Environmental Biology, Department of Biology, University of Minho, Braga, Portugal; ^5^ Facultad de Química, Departamento de Bioquímica, Universidad Nacional Autónoma de México, Ciudad de México, Mexico; ^6^ Instituto Politécnico Nacional, Centro de Biotecnología Genómica, Reynosa, Mexico

**Keywords:** transcriptomic, fusariosis, histology, SEM, lignin, ddPCR

## Abstract

**Introduction:**

The fungal pathogen *Fusarium verticillioides* (Sacc.) Nirenberg (*Fv*) causes considerable agricultural and economic losses and is harmful to animal and human health. *Fv* can infect maize throughout its long agricultural cycle, and root infection drastically affects maize growth and yield.

**Methods:**

The root cell wall is the first physical and defensive barrier against soilborne pathogens such as *Fv*. This study compares two contrasting genotypes of maize (*Zea mays* L.) roots that are resistant (RES) or susceptible (SUS) to *Fv* infection by using transcriptomics, fluorescence, scanning electron microscopy analyses, and ddPCR.

**Results:**

Seeds were infected with a highly virulent local *Fv* isolate. Although *Fv* infected both the RES and SUS genotypes, infection occurred faster in SUS, notably showing a difference of three to four days. In addition, root infections in RES were less severe in comparison to SUS infections. Comparative transcriptomics (rate +*Fv*/control) were performed seven days after inoculation (DAI). The analysis of differentially expressed genes (DEGs) in each rate revealed 733 and 559 unique transcripts that were significantly (P ≤0.05) up and downregulated in RES (+*Fv*/C) and SUS (+*Fv*/C), respectively. KEGG pathway enrichment analysis identified coumarin and furanocoumarin biosynthesis, phenylpropanoid biosynthesis, and plant-pathogen interaction pathways as being highly enriched with specific genes involved in cell wall modifications in the RES genotype, whereas the SUS genotype mainly displayed a repressed plant–pathogen interaction pathway and did not show any enriched cell wall genes. In particular, cell wall-related gene expression showed a higher level in RES than in SUS under *Fv* infection. Analysis of DEG abundance made it possible to identify transcripts involved in response to abiotic and biotic stresses, biosynthetic and catabolic processes, pectin biosynthesis, phenylpropanoid metabolism, and cell wall biosynthesis and organization. Root histological analysis in RES showed an increase in lignified cells in the sclerenchymatous hypodermis zone during *Fv* infection.

**Discussion:**

These differences in the cell wall and lignification could be related to an enhanced degradation of the root hairs and the epidermis cell wall in SUS, as was visualized by SEM. These findings reveal that components of the root cell wall are important against *Fv* infection and possibly other soilborne phytopathogens.

## 1 Introduction

*Fusarium* rots in maize (*Zea mays* L.) are caused by several *Fusarium* species (Leyva-Madrigal et al., 2015; Okello et al., 2019). These serious fungal diseases decrease maize production worldwide by affecting yield, plant growth, and/or seed quality mainly through the production of fumonisin or deoxynivalenol mycotoxins (Lizárraga-Sánchez et al., 2015; Hao et al., 2022). *F. graminearum* Schwabe and *F. verticillioides* (Sacc.) Nirenberg were the most predominant species reported in agriculture fields in Germany and New Zealand (Hussein et al., 2003; Görtz et al., 2008). *Fv* has also been detected in Brazil (de Sousa et al., 2022), China (Qiu et al., 2015), France (Atanasova-Penichon et al., 2014), and Mexico (Leyva-Madrigal et al., 2015). Mexico has high maize yield losses from *Fusarium* rot (González Huerta et al., 2007; Briones-Reyes et al., 2015; Lizárraga-Sánchez et al., 2015). For example, in Sinaloa State; the main maize producer reported a harvest of 5.5 million tons collected in the 2021 agricultural cycle (SIAP, 2021). These maize fields can exhibit *Fusarium* infection levels of up to 84% (Apodaca-Sánchez and Quintero-Benítez, 2008; García Pérez et al., 2012), with some maize fields reporting losses of 100% (personal communication from local farmers). In addition to these losses, the mycotoxins produced by *Fv* on maize grains are potentially harmful to human and animal health when consumed (Ross et al., 1990; Riley et al., 2006; Riley et al., 2015; Schrenk et al., 2022).

One current strategy to prevent the incidence of fungi is the application of chemical fungicides (Bashir et al., 2018). However, this practice can also be harmful to the environment and public health (Dangond Araujo and Guerrero Dallos, 2006). The breeding of maize-resistant genotypes is therefore an ideal and environmentally safe solution to address this problem. However, conventional breeding for disease resistance based on phenotypic characterization requires observing symptoms and screening large numbers of plants, making this approach expensive, time-consuming, and susceptible to misinterpretation (Haile et al., 2020). Massive sequencing technologies (“omics”) can provide tools to make this process more efficient, through marker-assisted selection (Haile et al., 2020; Wang et al., 2020) or the characterization and cloning of resistance genes (Steuernagel et al., 2016; Arora et al., 2019; Kim et al., 2021). In addition, omics strategies could help to understand plant biological processes such as growth, development, and abiotic and biotic stress resistance (Agrawal et al., 2015). As a first step, relevant gene expression can be selected from a comparative transcriptomics analysis between contrasting phenotypes, such as resistance (RES) *vs.* susceptible (SUS) maize phenotypes to pathogen infection. Indeed, the maize response to several pathogens, such as *F. graminearum*, *Cercospora zeae-maydis*, and *F. virguliforme* has already been studied using transcriptomics analysis (Liu et al., 2016; Yu et al., 2018; Baetsen-Young et al., 2020). These studies show that these pathogens trigger common defense mechanisms in resistant lines, including the silencing of auxins, salicylic acid accumulation, activation of reactive oxygen species pathways, and the expression of transcriptional factors such as PR-genes and R-genes, which are involved in the plant–pathogen interaction. Genes implicated in cell wall reinforcement and the modification of silk and kernels have also been reported (Kebede et al., 2018; Zhou et al., 2020). Recently, Wang et al. (2022) analyzed the fungal pathogenic response to maize-susceptible B73 and reported the importance of diterpenoids, phenylpropanoid, and lignin (cell wall) pathways during disease resistance in ear rot. Specifically, *Fv* can cause rot throughout the life cycle of maize, from seed to post-germination to the reproductive stage, in ears and kernel tissue (Wu et al., 2011; Roman et al., 2020). In the case of root rot, *Fv* colonizes the roots *via* injuries or wounds generated by the emergence of lateral roots or root growth (Wu et al., 2011). This fact is important since this phytopathogen is ubiquitous in the soil (Wu et al., 2011). Moreover, the root cell wall acts as the first barrier against infection before the fungus spreads to other plant tissues. This current study examines the transcriptomic response of two contrasting maize phenotypes (pathogen resistant, or RES, and pathogen susceptible, or SUS) to root *Fv* infection. The aim of this was thus to determine if the cell walls in the maize inbred lines have any role in *Fv* infection.

## 2 Materials and methods

### 2.1 Biological material

The inbred maize lines SUS (IL09) and RES (IL02) were used, whose contrasting responses to *Fv* root rot were previously evaluated (Román, 2017; Roman et al., 2020). The highly virulent *Fv* strain DA42 was previously characterized by Leyva-Madrigal et al. (2015) and was used to conduct the infection experiments.

### 2.2 Seedling root rot

Previously described protocols were used for seedling preparation (Warham et al., 1996; Román, 2017; Roman et al., 2020). Briefly, the seeds were superficially disinfected by sonication (2.8 L Ultrasonic Bath, Fisher Scientific) in sterile distilled water with Tween 20 (five drops of Tween 20/100 ml of distilled water) for 5 min. Subsequently, the seeds were immersed in 1.5% (V/V) sodium hypochlorite at 52°C for 20 min in a thermobath (FE-377, Felisa) and rinsed three times in sterile distilled water under sterile conditions in a biological safety cabinet (Herasafe KS, Thermo Scientific). The fungus was cultured in Spezieller Nährstoffarmer agar medium (SNA) (Leslie and Summerell, 2007) with 1 cm^2^ filter paper at 25 ± 2°C for 14 days. Conidia were harvested by adding 5 ml of sterile saline solution (0.8% NaCl) to the culture medium with gentle shaking. The conidia working aqueous solution was prepared at a final concentration of 1 × 10^6^ conidia/ml by quantification in a Neubauer chamber (cat. No. 3110, Hausser Scientific, USA) using a light microscope (B-383-M11, Optika, Italy). Disinfected seeds were immersed for 5 min in the conidia work solution, whereas control seeds were immersed in sterile water. Subsequently, 10 seeds were distributed, each 2 cm thick, on sterile Kraft paper (40 cm length × 20 cm width), moistened with sterile water, rolled up, and placed in plastic bags. The seeds germinated in a 16:8 h light:dark photoperiod for seven to 10 days at 25°C. The humidity of the rolls was maintained by irrigating them with 15 ml of water every 24 h. Visual records and photographs were taken every day with a stereo microscope (model M205FA, Leica, Germany).

### 2.3 From RNA extraction to *in silico* analysis

Root RNA was extracted at seven days old after seed germination for both RES (uninfected and infected) and SUS (uninfected and infected) genotypes. Total RNA extraction was performed using the RNeasy^®^ Plant Mini Kit (Qiagen, Cat. No. 74904), according to the manufacturer. For library preparation and sequencing, 500 ng of RNA were used as input material for each RNA sample preparation. Twelve libraries (three independent biological replicates) per analyzed condition (uninfected and infected plants) from the SUS and RES genotypes were generated. The TruSeq RNA Sample Preparation Kit (Illumina, Cat. N°RS-122-2002) was used following the manufacturer’s recommendations, and index codes were utilized to identify each sample independently. The libraries were sequenced on the NextSeq 500 platform (Illumina) using the 2 × 150 bp paired-end sequencing protocol in the facilities of LANGEBIO (langebio.cinvestav.mx). The bioinformatic analysis was conducted on the OOREAM server of IPN, CIIDIR Sinaloa. The quality of reads was examined with FastQC v0.11.7 (www.bioinformatics.babraham.ac.uk/projects/fastqc/) before and after the trimming process. Raw reads were filtered with Trimmomatic 0.36 (Bolger et al., 2014) to eliminate adapters, low-quality reads (Q <20), and short reads (<50 bp). Trimmed reads were pseudo-aligned with Kallisto v0.46.2 (Bray et al., 2016) to the reference transcriptome of *Z. mays* cv. B73 v5 (www.maizegdb.org). The transcript abundance files were imported into R v4.2.2 with the package tximport v1.26.1, and then differential expression analysis was conducted with the package DESeq2 v1.38.3 (Love et al., 2014). Differentially expressed genes (DEGs) were defined as those genes with an adjusted P-value <0.05 and a log2 fold change ±1. DEGs were subjected to KEGG (Kyoto Encyclopedia of Genes and Genomes) and GO (Gene Ontology) enrichment analyses with the packages clusterProfiler v4.6.2 (Wu et al., 2021) and REVIGO (Supek et al., 2011), respectively.

### 2.4 Microscopy analyses

Primary root tissue close to the seed (the first 0.5 cm) was harvested for light microscopy and scanning electron microscopy (SEM) studies. For light microscopy, tissues were fixed in 96% ethyl alcohol and stored at 4° C. Tissues were then placed in a histocassette and gradually dehydrated in ethyl alcohol. Subsequently, they were placed in a 1:1 solution of ethyl alcohol and 100% xylol (Spintissue Processor STP120 and Histo Star Embedding Centre, Thermo Scientific). The dehydrated samples were embedded in Histoplast (cat. no. 22900700, Fisherbrand), and 5 μm-thick cross-sections were cut with a microtome (Microm HM 340E, Thermo Scientific) and incubated in a drying oven (DX402, Yamato) at 60° C for 30 min, then placed in xylene for 2 min to remove paraffin. Tissue samples were then rehydrated in 1× PBS and visualized for endogenous fluorescence by epifluorescence microscopy (DIM6000, Leica, Germany). For SEM, the samples were treated as previously described by Olivares-Garcia et al. (2020).

### 2.5 Cell wall gene expression

RNA was extracted from RES (C and +*Fv*) and SUS (C and +*Fv*). Each sample was integrated with the root tissue from 20 plants, and the tissue was macerated in liquid nitrogen. Total RNA isolation was performed using TRIzol™ (15596018, Ambion, CA) according to the manufacturer’s specifications. The cDNA was synthesized using 1,000 ng of RNA treated previously with DNAse (AM2222, Invitrogen) using the Super Script™ IV First Strand Synthesis System kit (18091050, Invitrogen) according to the manufacturer’s protocol. A ddPCR (droplet digital PCR) was performed according to the manufacturer’s instructions; briefly, the Supermix for EvaGreen Qx200TM was used, and the reaction was carried out in a final volume of 20 µl with final concentrations of 1× Qx200, 100 nM for each primer, and 50 ng of cDNA. The drops were generated in droplet generator equipment, and these were recovered and transferred to a 96-well ddPCR plate (BIO-RAD, USA). The PCR protocol consisted of one cycle of enzyme activation at 95°C for 5 min; 40 cycles of denaturation at 95°C for 30 s; alignment and extension at 60°C for 1 min with an increase of 2°C every second; and one signal stabilization cycle consisting of 4°C for 5 min and then 90°C for 5 min. And in the end, a storage temperature of 4°C. The reaction was carried out in a T100™ thermal cycler (BIO-RAD, USA). At the end of the protocol, the drops were read on the QX200™ Droplet Reader (BIO-RAD, USA). Three technical replicas for each independent biological replicate were used for the quantification assay. The result was reported in copies per µl (copies/µl), and the statistical analysis and the graphics were performed by OriginPro 8.5 software (developed by OriginLab Corporation, Northampton, USA), using the statistical Tukey test with a significance of 0.05. Primers were designed for peroxidases (XM_020546021, Fw-CCGAGGACATCATCAAGCAA and Rv-GAGTTGATGAGGATGGAGCC), cellulose synthases (XM_035960774.1, Fw-CGCTGGATTTGACGACGA and Rev-AGGAACACCACCATACTCCA), and expansin (EU960208.1, Fw-TTTTCTCCTCCCCATCCAGT and Rev-CTTCACGGAGGCACTTAACA) involved in cell wall. The design was carried out using the Primer3plus software with parameters suggested for the dd PCR.

### 2.6 Statistical analyses

Raw data from the microscopy studies were analyzed to determine if they differed from a normal distribution (Shapiro–Wilk test). Variables that conformed to parametric assumptions were analyzed using one-way analysis of variance (ANOVA) and Duncan’s means test (α <0.01); those that did not were analyzed using the non-parametric Kruskal–Wallis test and Dunn’s pairwise comparison test. The experiment was repeated three times independently, with three replicates (n = 15) for microscopy analyses. All statistical analyses were conducted in R v4.2.2. Origin v8.5.1 and CorelDraw v20.0.0.633 were used to generate graphs and figures, respectively.

## 3 Results

### 3.1 RES and SUS genotypes display macroscopic differential responses to *Fv* infection

**Figure 1:**
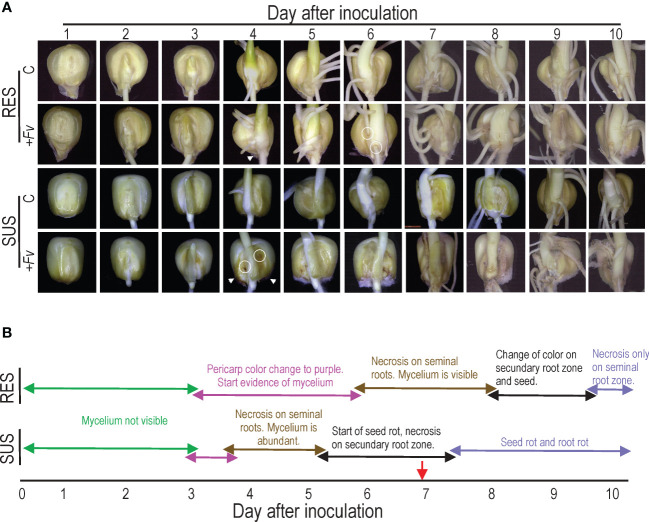
Time-course observations reveal that *Fusarium verticillioides* infection spreads faster in SUS than RES. **(A)** Resistant (RES) inbred lines and susceptible (SUS) inbred lines that were either uninfected **(C)** or infected with *F*. *verticillioides* (+*Fv*) were observed over 10 days. Seeds were infected with an *F*. *verticillioides* conidia concentration of 1 × 10^−6^ conidia/ml and recorded daily. The arrowheads indicate the onset of *F*. *verticillioides* mycelia growth, whereas the circles represent the start of necrotic spots. **(B)** Schematic representation of changes in seeds and roots of RES and SUS genotypes. The red arrow indicates the sample time used for subsequent studies.

An infection time course was performed to investigate the *Fv* infection (+*Fv*) process in RES and SUS genotypes and to determine the optimal day to visualize sample root tissue for transcriptomics analysis (**Figure 1**). The color of the pericarp turned purple in the infected seeds of RES and SUS, and the *Fv* mycelium was visualized by stereomicroscopy three days after seed inoculation (DAI). However, the mycelium was visible until four DAI (**Figure 1A**, arrowheads). Mycelium abundance was more evident in SUS than in RES, and the pericarp displayed necrotic spots only in SUS (**Figure 1A**, circle). At four to five DAI, small necrotic spots were visible on SUS seed roots, and seed rot commenced, whereas necrotic spots were observed on RES between six and seven DAI. Necrotic spots on secondary roots began at five DAI in SUS. In contrast, RES only showed a color change up to eight DAI in the same tissue. SUS seed rot began at five or six DAI, becoming evident at eight DAI (**Figure 1B**). Interestingly, seed rot in RES was observed at 12 DAI (data not shown), demonstrating that seven DAI is the most suitable time for the analyses.

### 3.2 Differential expression analysis from RES (+*Fv*/C) and SUS (+*Fv*/C) genotypes

Gene expression during *Fv* root infection in RES and SUS genotypes was analyzed by RNA-Seq. Since *Fv* induces necrosis in infected tissues, seven DAI were selected for RNA isolation from root tissue to preserve the highest possible RNA integrity and quality. Only samples with RNA integrity numbers between 7.9 and 9.9 were considered for library construction and sequencing. Three biological replicates for each condition were obtained, but one atypical replicate per condition was removed according to principal component analysis (**Supplementary Figure S1A**). On average, 83.2 and 76.2 million trimmed reads per library were kept for RES (C and +*FV*) and SUS (C and +*FV*) genotypes, respectively (**Supplementary Table S1**). The differential expression analysis in response to +*Fv* infection (**Supplementary Tables S2**, **S3** for RES and SUS, respectively) showed 798 and 624 DEGs for RES and SUS genotypes, respectively. From those, 733 and 559 DEGs were exclusive for RES and SUS, respectively, and only 65 DEGs were common in both genotypes. In RES, 294 DEGs (40%) were upregulated and 439 DEGs (60%) were downregulated. While in SUS, 258 DEG (46%) were upregulated and 301 DEGs (54%) were downregulated (**Supplementary Figure S2**). The expression profiles of DEGs for RES and SUS genotypes were visualized in heatmaps (**Supplementary Figures S1B, C**), showing similar profiles between the two replicates of each condition.

### 3.3 KEGG and GO terms analysis

**Figure 2:**
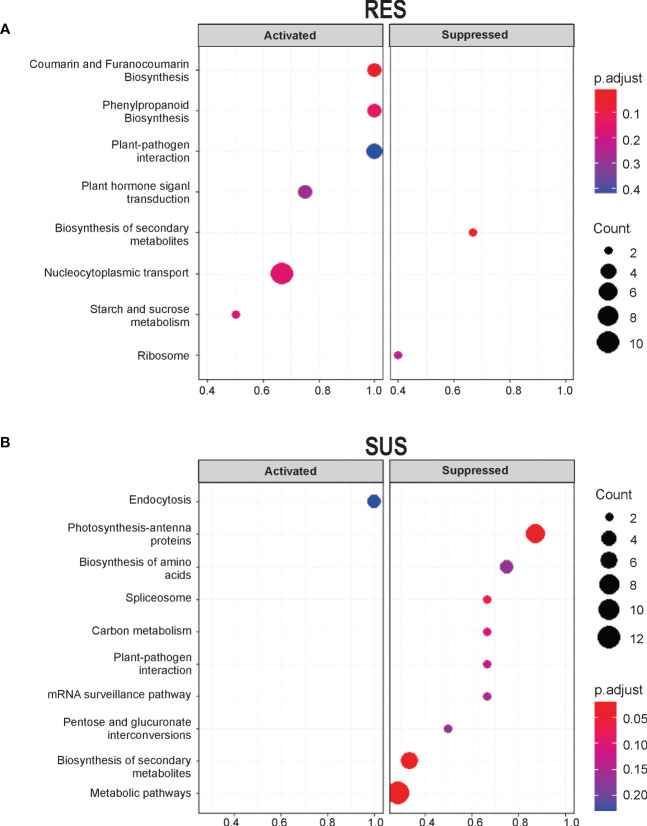
KEGG pathway enrichment analysis of unigenes shows activation in RES and suppression in SUS at seven DAI. KEGG enrichment pathways of the DEGs were exclusively detected in **(A)** RES (+*Fv*/C) and **(B)** SUS (+*Fv*/C). Significantly enriched pathways with a corrected p-value (q value) <0.05 are shown. The number indicates the size of the dot, describing the number of unigenes enriched in the pathway. The color bar represents the RF value and indicates the significance of the enrichment.

To identify pathways involved in *Fusarium* infection, we performed a KEGG enrichment analysis. At a p-value threshold of ≤0.05, several pathways were highlighted in the two comparisons (**Figure 2** and **Table 1**). In general, RES genotype showed metabolic pathway enrichment to produce specific metabolites, i.e., coumarin and furanocoumarin, phenylpropanoids, and transcripts related to plant-pathogen interactions (GeneRatio = 1). In contrast, only SUS showed positive enrichment for transcripts involved in membrane rearrangements (endocytosis), and a negative enrichment for pathways involved in photosystems and the plant–pathogen interaction pathway, among others (GeneRatio >0.8). Specific transcripts involved in the KEGG analysis (**Supplementary Table S4**) indicate that several transcripts are related to cell wall proteins (pectinesterase, phenylalanine ammonia-lyase, beta-amylase, and peroxidase). In addition, the RES genotype exhibits enrichment in the phenylpropanoid biosynthesis pathway, which contains transcripts related to cell wall biogenesis.

**Table 1:** Uniprot ID identified in the KEGG enrichment analysis (p-value <0.5).

	Transcript_ID	Uniprot	log2 FoldChange	Protein names
**RES**	Zm00001eb105040_T005	A0A1D6EVQ9	21.645	Trehalose 6-phosphate phosphatase (EC 3.1.3.12)
	Zm00001eb003440_T003	B6SYP0*	9.998	Beta-amylase (EC 3.2.1.2)
	Zm00001eb304070_T002	A0A1D6HWI3*	6.606	UDP-glycosyltransferase 76C1
	Zm00001eb014700_T001	Q8LT19	4.331	Nicotianamine synthase (EC 2.5.1.43)
	Zm00001eb014680_T001	A0A1D6K0A7	4.127	Nicotianamine synthase (EC 2.5.1.43)
	Zm00001eb017950_T001	A0A1D6K433*	2.933	Peroxidase (EC 1.11.1.7)
	Zm00001eb417380_T001	B6TJX6	2.711	PTI1-like tyrosine-protein kinase 3 (Pto kinase interactor 1)
	Zm00001eb036940_T001	Q9ZQY3	2.461	Pyruvate dehydrogenase E1 component subunit beta (EC 1.2.4.1)
	Zm00001eb129910_T002	B6T7D0*	2.402	PEROXIDASE_4 domain-containing protein
	Zm00001eb299370_T001	B4FVP5	2.229	Pathogenesis related protein4
	Zm00001eb299370_T001	O82086	2.229	Pathogenesis related protein-1
	Zm00001eb201390_T001	B6SIR9	2.185	1-aminocyclopropane-1-carboxylate oxidase
	Zm00001eb348950_T001	K7VFH6*	2.049	Peroxidase (EC 1.11.1.7)
	Zm00001eb058470_T005	B6TGM8	1.332	Osmotic stress/ABA-activated protein kinase (Ser/thr-protein kinase SAPK8)
	Zm00001eb065720_T001	A0A096TI73	−1.227	CASP-like protein 1
	Zm00001eb435160_T001	P25459	−8.550	30S ribosomal protein S18, chloroplastic
	Zm00001eb435160_T001	A0A5P8KLE7	−8.550	30S ribosomal protein S18, chloroplastic
**SUS**	Zm00001eb171040_T003	K7UAQ8	12.336	ADP-ribosylation factor GTPase-activating protein AGD10
	Zm00001eb303500_T001	B4FMJ0	3.057	Charged multivesicular body protein 4b
	Zm00001eb061610_T001	B4FGC0	2.641	PKS_ER domain-containing protein
	Zm00001eb210570_T001	B4FK00	2.641	Putative alcohol dehydrogenase superfamily protein
	Zm00001eb171040_T003	A0A3L6F1T5	2.153	ADP-ribosylation factor
	Zm00001eb326780_T007	A0A3L6E405	1.899	3-hydroxyacyl-[acyl-carrier-protein] dehydratase
	Zm00001eb301540_T001	B6TI69	1.892	Tryptophan synthase (EC 4.2.1.20)
	Zm00001eb018300_T004	O04981	1.832	Cystathionine gamma-synthase (EC 4.2.99.9)
	Zm00001eb331200_T001	B6T148	1.669	Calmodulin
	Zm00001eb073670_T001	A0A1D6E2H5	1.440	Enoyl-[acyl-carrier-protein] reductase [NADH] chloroplastic
	Zm00001eb199330_T003	B4FMA8	1.094	RNA-binding (RRM/RBD/RNP motifs) family protein (SNF2 transcription factor)
	Zm00001eb357740_T001	B4FNR1	−6.002	Chlorophyll a-b binding protein, chloroplastic
	Zm00001eb357740_T001	B4G143	−6.002	Chlorophyll a-b binding protein, chloroplastic
	Zm00001eb357740_T001	B6STN4	−6.002	Chlorophyll a-b binding protein, chloroplastic
	Zm00001eb357740_T001	P12329	−6.002	Chlorophyll a-b binding protein 1, chloroplastic (LHCII type I CAB-1) (LHCP)
	Zm00001eb295170_T005	C0P4C8	−8.788	Serine/threonine protein phosphatase 2A regulatory subunit
	Zm00001eb325410_T002	B4F9W3	−10.085	Chlorophyll a-b binding protein, chloroplastic
	Zm00001eb325410_T002	B6T892	−10.085	Chlorophyll a-b binding protein, chloroplastic
	Zm00001eb325410_T002	B6TKL9	−10.085	Chlorophyll a-b binding protein, chloroplastic
	Zm00001eb325410_T002	B4F9W3	−10.085	Chlorophyll a-b binding protein, chloroplastic
	Zm00001eb325410_T002	B6T892	−10.085	Chlorophyll a-b binding protein, chloroplastic
	Zm00001eb325410_T002	B6TKL9	−10.085	Chlorophyll a-b binding protein, chloroplastic
	Zm00001eb185030_T001	B6TS21	−10.354	Succinate–CoA ligase [ADP-forming] subunit beta, mitochondrial (EC 6.2.1.5)
	Zm00001eb003440_T003	B6SYP0*	−10.588	Beta-amylase (EC 3.2.1.2)
	Zm00001eb200350_T001	Q208N4	−21.646	Calcium and calcium/calmodulin-dependent serine/threonine-proteinkinase
	Zm00001eb130340_T004	B4FFT5	−21.718	Lysophospholipid acyltransferase LPEAT1
	Zm00001eb345420_T005	K7UTZ2	−21.738	Spliceosome RNA helicase BAT1 isoform 1
	Zm00001eb247650_T001	A0A1D6HDL9*	−23.144	Phenylalanine ammonia-lyase (EC 4.3.1.24)
	Zm00001eb291670_T003	C0PDB0	−23.181	Phosphoglycerate kinase (EC 2.7.2.3)
	Zm00001eb130500_T001	A0A3L6FHM0*	−23.870	Pectinesterase (EC 3.1.1.11)
	Zm00001eb130500_T001	B8A2X5*	−23.870	Pectinesterase (EC 3.1.1.11)
	Zm00001eb215080_T002	C0PFQ7	−24.338	Sulfate adenylyltransferase (EC 2.7.7.4)
	Zm00001eb105040_T005	A0A1D6EVQ9	−32.664	Trehalose 6-phosphate phosphatase (EC 3.1.3.12)

*Indicates transcripts related with cell wall proteins.

**Figure 3:**
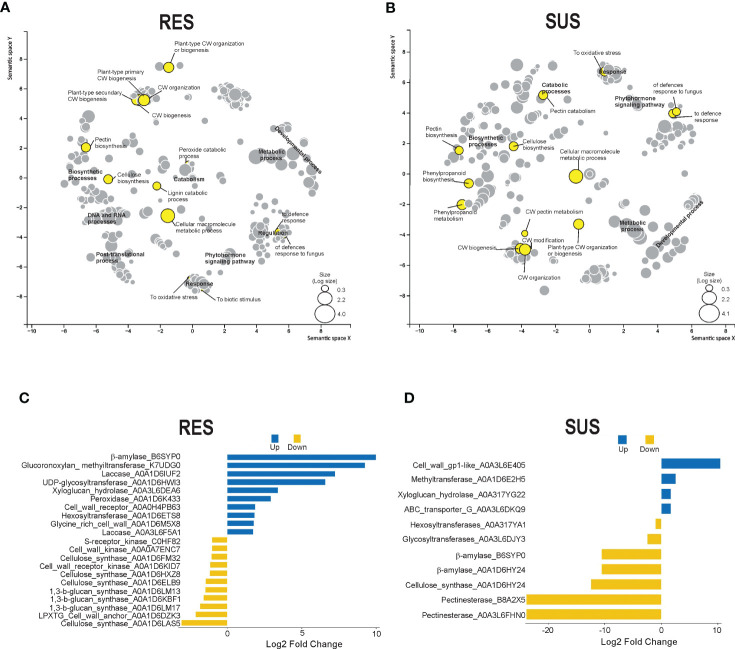
DEG GO enrichment and cell wall-related protein analysis. Scatterplot showing the enrichment of biological processes in abundance for **(A)** RES (+*Fv*/C) and **(B)** SUS (+*Fv*/C) in the transcriptome using REVIGO. The circle size is proportional to the GO DEG abundance. Cell wall proteins were obtained from DEG identification in RES **(C)** and SUS **(D)**. The blue and yellow bars represent up and down log2 fold changes in gene expression.

A more detailed analysis of the DEG was conducted to identify the GO terms in abundance (**Figures 3A, B**). This analysis made it possible to identify clustered transcripts related to proteins in the same pathways, which were also previously detected in KEGG enrichment analysis. In general, GO term abundance analysis revealed transcripts related to secondary metabolism, phenylpropanoids, lignin, cell wall processes, plant–pathogen interactions, and hormone signal transduction in maize RES and SUS genotypes. Interestingly, some cell wall transcripts related to cell wall organization biogenesis, cell wall organization, cell wall biogenesis, pectin biosynthesis, and cellulose biosynthesis were also detected. Attention was therefore focused on transcripts related to the cell wall that were identified in the transcriptomes of both maize genotypes.

### 3.4 Cell wall-related transcripts analysis

Twenty-one transcript IDs in the RES genotype and eleven transcript IDs in the SUS genotype were identified as cell wall-related transcripts (**Figures 3C, D**). Some of these transcripts were also identified in the previous KEGG enrichment analysis, specifically peroxidase (A0A1D6K433), UDP-glycosyltransferase (A0A1D6HWI3), beta-amylase (B6SYP0), and pectinesterases (A0A3L6FHM0 and B8A2X5). The RES genotype shows a higher number of expressed cell wall-related transcripts as compared to the SUS genotype. RES genotype also has a high percentage (47%) of cell wall transcripts that are highly expressed in relation to the control condition, whereas SUS genotype shows a high percentage (64%) of downregulated cell wall-related transcripts relative to the control condition (**Figures 3C, D**). DEG transcripts putatively related to lignin metabolism, including peroxidases and laccases, were upregulated (1.7 to 7.2 log2 fold change) in RES genotype, whereas the phenylalanine ammonia-lyase transcript was surprisingly downregulated (−23.1 log2 fold change) in the SUS genotype. Interestingly, another cell wall remodeling enzyme, beta-amylase, was upregulated (9.9 log2 fold change) in the RES genotype, whereas it was downregulated (−10.5 log2 fold change) in the SUS genotype. In addition, pectinesterases were strongly downregulated (−23.8 Log2 FC) in the SUS genotype. Finally, beta-amylase and several pectinesterases were strongly reduced in the SUS genotype, suggesting cell wall disorganization in this genotype, whereas the RES genotype displayed more transcriptional activity than the SUS genotype among genes related to cell wall dynamics.

### 3.5 The cell wall of maize roots is stronger in the RES genotype than in the SUS genotype

**Figure 4:**
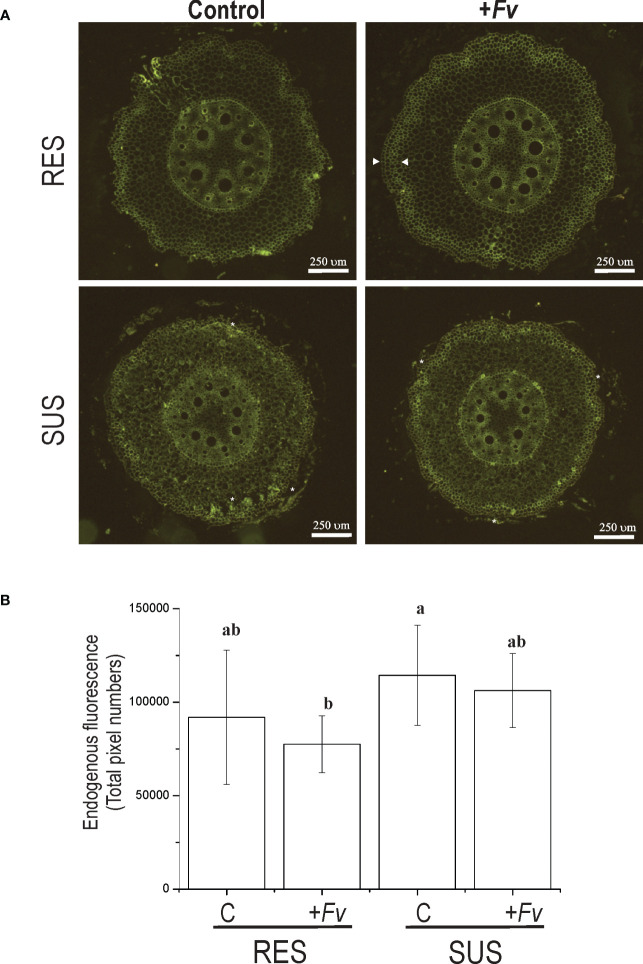
Endogenous fluorescence emission is increased in sclerenchymal cells of the hypodermis zone in RES roots infected with *Fusarium verticillioides*. **(A)** Cross-sections of RES roots and SUS roots that were either uninfected (control) or infected (+*Fv*) by *Fusarium verticillioides* were excited by a UV–visible lamp and recorded in the green emission range. **(B)** The measurement of endogenous fluorescence emission cross-sections is represented as the total pixel number along the y-axis.

**Figure 5:**
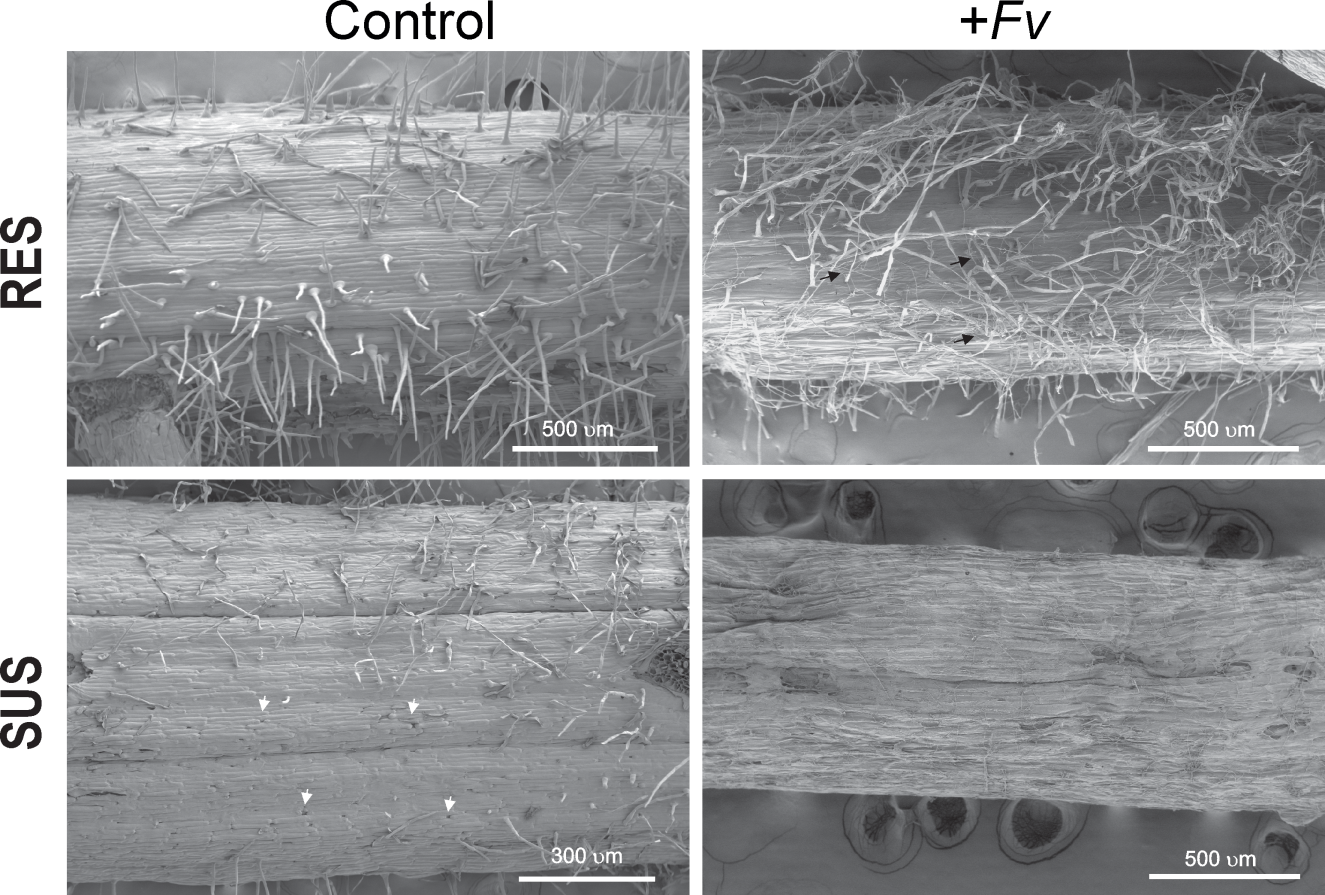
The susceptibility of SUS is related to cell wall weakness. Scanning electron microscopy of the primary root close to the pedicel of RES and SUS that was either uninfected (control) or infected (+*Fv*) by *Fusarium verticillioides*.

To examine the potential role of the cell wall in maize roots during *Fv* infection, histological sections were made from the first 5 mm of the primary root at the pedicel zone and visualized by endogenous fluorescence (**Figure 4**). The spatial distribution of fluorescence was slightly higher in the SUS genotype than in the RES genotype (**Figure 4B**). However, the RES genotype fluorescence was increased in the first cellular layers of the epidermis–hypodermis zone during *Fv* infection (**Figure 4A**, arrowheads). In contrast, the SUS genotype presented a more homogenous fluorescence along with more fragile cellular tissues during histological manipulation (**Figure 4A**, asterisks), which could compromise the epidermis–hypodermis integrity during *Fv* attack. The average endogenous fluorescence decreased in both RES and SUS genotype tissues during *Fv* infection in comparison to the control, although no statistical differences were observed. The SUS genotype displayed a higher fluorescence in both control and infected samples than the RES genotype (**Figure 4B**). To study the first cellular layer (epidermis), which is the first site to be targeted by *Fv* attacks, the primary roots of uninfected and infected tissues were observed by SEM (**Figure 5**). In the uninfected conditions, RES root hairs were turgid and there was a homogenous distribution of epidermis cells, whereas the root hairs in SUS were not turgid, and SEM of SUS epidermis cells revealed small holes between cell–cell junctions, probably due to an abnormal distribution in the cells (**Figure 5**, arrowheads). In infected roots, SUS root hairs and the epidermis cell wall were degraded by *Fv* infection, whereas in RES, the *Fv* mycelium could be visualized between the root hairs (**Figure 5**, arrows), and cells were slightly turgid but not collapsed. Taken together, these results suggest that the cell walls of the first cellular layers in maize roots play an important role during *Fv* infection. Lignin content and confocal microscopy analysis (manuscript in preparation) have revealed a spatial distribution of lignin (stained with basic fuchsin) similar to that observed by endogenous fluorescence, which was correlated with the increased signal in sclerenchyma cells (hypodermis). These findings suggest that the cell wall of the first cell layers at the epidermis–hypodermis zone may have an active role against *Fv* infection due to the strengthening of the cell wall by lignification.

### 3.6 The resistance of the root cell wall is correlated with higher gene expression levels of cell wall-related genes in RES than SUS

**Figure 6:**
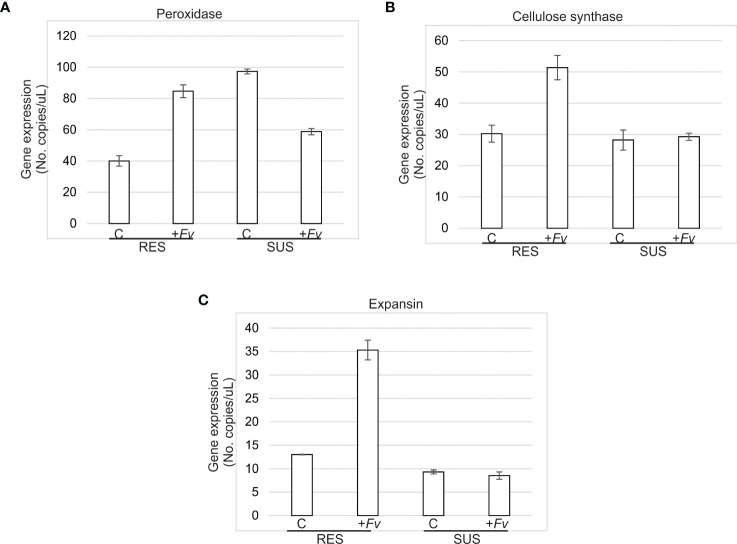
Cell wall-related gene expression was more upregulated in RES than SUS during *Fv* infection. **(A)** Peroxidase, **(B)** Cellulose synthase, and **(C)** Expansin gene expression was quantified in RES and SUS roots uninfected (control) and infected (*+Fv*) at 7 days after *Fusarium verticillioides* seed inoculation by droplet digital PCR (ddPCR).

The gene expression levels of the three-cell wall-related genes evaluated were higher in RES than in SUS during *Fv* infection (**Figure 6**). In the control, Prx gene expression was higher in SUS than RES; however, the expression has increased in RES, contrary to SUS, where its expression was downregulated in roots infected by *Fv*. For Cesa, in both RES and SUS genotypes, expression was closed, whereas Cesa expression was stimulated by *Fv* infection; suppressively, SUS root cells did not change their expression levels. This same behavior in gene expression was observed when expansin expression was evaluated. These observations suggest that the root cell wall in RES has a better dynamic to protect against *Fv* infection.

## 4 Discussion

To ensure food security, breeders must focus on making crop plants more resilient to abiotic and biotic stresses as well as diseases. *Fv* is a phytopathogen that decreases maize production by affecting plant growth and grain quality (Lizárraga-Sánchez et al., 2015). *Fv* secretes mycotoxins that cause neural tube defects, cancer, craniofacial anomalies, and other birth defects in humans and animals (Marasas et al., 2004; Riley et al., 2006; Riley et al., 2015). One strategy to bring down this pathogen and its harmful effects from the food chain is to develop maize hybrids that are resistant to *Fv* rots (Roman et al., 2020); studies of contrasting phenotypes in maize could also help to understand plant mechanisms used for defense or resistance against the *Fusarium* infection process.

Here, we conducted a comparative root study involving transcriptomics, cellular analyses, and ddPCR in RES and SUS maize genotypes infected with *Fv*. In general, there are important differences in *Fv* infection between RES and SUS genotypes (**Figure 1**), especially at the beginning. *Fv* colonization and necrosis spread faster in SUS than in RES, notably with a difference of 3–4 days. Necrosis symptoms in the SUS genotype appeared at five DAI. Similarly, the study of *Fv* infection *via* GFP expression in susceptible maize revealed discolored seedlings and infected radicle symptoms 24 h after inoculation and visible seed rot symptoms (purple color in roots) at five DAI (Gai et al., 2018). These differences could be due to differences in inoculation methods, maize genotype, and/or *Fv* strain.

Our results indicate that the RES genotype increased the number of cellular layers or cell wall thickness in the hypodermis zone during *Fv* infection (**Figure 4**). Brown spots were observed on the root epidermis but not in vascular tissues, although the endogenous fluorescence was slightly higher in the SUS genotype with a wider distribution. Similar results were observed in contrasting cotton cultivars, where the *Fusarium* infection caused vascular browning in susceptible cultivars but not in resistant cultivars. When these tissues were observed by endogenous fluorescence emission, a more intense fluorescence was seen in the vascular tissue of susceptible cultivars (Zhu et al., 2022). Root hypodermis cells are formed from sclerenchymal cells, which have thick-walled, frequently lignified cell walls (Esau, 1965). Therefore, differences in *Fv* infection in these genotypes could be related to cell wall modifications as well as gene expression.

Regarding expression analysis, the libraries obtained 733 and 559 unique DEGs for RES (rate C/+*Fv*) and SUS (rate C/+*Fv*), respectively (**Supplementary Figure S2**). These numbers are quite low in comparison to other studies of contrasting maize genotypes. For example, 2,250 and 2,442 DEGs were previously obtained for resistance and susceptibility to *Fv* ear rot, respectively (Lanubile et al., 2014). These differences in transcriptomic number could be due to the studied tissues and/or genotypes. In the current study, DEG analysis revealed that the RES genotype has 1.3-fold more DEGs than the SUS genotype, although a similar number of DEGs were up and downregulated in both genotypes. Similar results were obtained by Lanubile et al. (2014), with 73% and 82% of upregulated genes in *Fv* resistance and susceptibility to ear rot, respectively. KEGG analysis (**Figure 2**) shows that plant–pathogen interactions, phenylpropanoids, and secondary metabolism pathways are enriched in the RES genotype, whereas the SUS genotype shows negative enrichment for these pathways. Specific genes involved in the KEGG analysis show highly regulated proteins involved in cell wall modification. Resistance mechanisms, secondary metabolism, and phenylpropanoid metabolism were also represented in the DEG analysis conducted on contrasting maize genotypes during ear rot (Lanubile et al., 2014). In sugarcane, phenylpropanoid metabolism was enriched during *Fv* infection in the resistant variety (Wang et al., 2019). Phenylpropanoids are a large class of secondary metabolites that are implicated in physiological processes, including cell wall fortification by lignin during plant pathogen attack (Santiago et al., 2013; Yadav et al., 2020; Bauters et al., 2021). The GO terms abundance analysis (**Figure 3**) shows similar results to those obtained in the KEGG enrichment analysis, detailing several specific cell wall-related transcripts. These transcripts are related to cell wall organization biogenesis, pectin biosynthesis, and cellulose biosynthesis. Other authors have also reported the abundance of GO terms enriched in the response to stimuli and carbohydrate metabolic processes (Lambarey et al., 2020). The abundance of GO terms involved in cell wall modification has also been documented in contrasting sugarcane cultivars (Wang et al., 2019). Specifically, the current study showed that the more regulated cell wall transcripts (up and down) included beta-amylase, glucuronoxylan methyltransferase, peroxidases, laccase, phenylalanine ammonia lyase, and pectinesterases. Similarly, lignin, cellulose, callose, and pectin genes are differentially regulated in contrasting cultivars during the infection process (Wang et al., 2019). Gene quantification of cell wall-related genes showed higher expression in RES than SUS during *Fv* infection (**Figure 6**). This data supports the involvement of the cell wall as a physical barrier against *Fv* infection. The presence of lignin and other cell wall components can also help reduce the spread of fungal infections (Miedes et al., 2014). Lignin is a complex molecule that plays an important role in resisting fungal infection by mechanical and hydration resistance, in addition to producing antifungal molecules by lignin-derived phenolic compounds (Santiago et al., 2013; Yadav and Chattopadhyay, 2023). Another highly downregulated transcript encodes phenylalanine ammonia lyase (PAL). This key enzyme is at the beginning of the phenylpropanoid pathway and is therefore involved in the biosynthesis of phenol molecules such as lignin (Vanholme et al., 2019). In addition, PAL silencing increased the penetration of the fungus *Blumeria graminis f.* sp. *tritici* in wheat (Bhuiyan et al., 2009). Pectin is an important component of plant cell walls involved in cell-cell adhesion and cell wall porosity, as its alteration/modification affects the plant disease response, and pectinesterase activity helps to loosen and increase cell wall extensibility (Shin et al., 2021; Wan et al., 2021).

SEM analysis (**Figure 5**) revealed small holes between cell–cell junctions in the SUS epidermis cells and the degradation of root hairs under *Fv* infection. In contrast, RES root hairs became slightly turgid but did not degrade under *Fv* infection. Lignin content is higher (data not shown) in RES than in SUS, and lignin distribution between the first cell layers might be involved in strengthening the cell wall in the RES genotype. Fungi, including *Fv*, are more likely to infect and colonize roots with weak cell walls than roots with a high amount of lignin accumulation possible by cross-linking among arabinoxylans and phenols (Santiago et al., 2013; Lee et al., 2019). Gene expression supports our observations of the cell wall by SEM, in which the RES genotype exhibits stronger cell walls than the SUS genotype. This deterioration in the cell wall of the epidermis and degradation of root hairs could facilitate the entry of *Fv* and other pathogens. In addition, plant growth is also affected since the plant cannot take up (disrupt) water and soil nutrients, and sap flux is interrupted. Taken together, these results suggest that the root cell wall is the first target during *Fv* attack, and it could have a more relevant role in the RES genotype than in the SUS genotype. To our knowledge, this is the first report to use comparative transcriptomics and microscopy to study root RES *vs.* root SUS infected with *Fv* in maize genotypes.

## 5 Conclusion

We applied comparative RNA-Seq analysis and cellular biology studies to examine how contrasting maize ILs respond to *Fv* infection, providing the first evidence of a maize root mechanism involved in pathogen infection. Our results demonstrate that the RES genotype is resistant to *Fv* due to the increased lignin content in the first cellular layers and that this epidermis–hypodermis zone is not easily degraded, as it is in the SUS genotype. To our knowledge, this is the first evidence of any root lignin mechanisms involved in the soilborne *Fv* pathogen infection process, and it is possible that other soilborne pathogens use similar mechanisms of infection.

## Data availability statement

The datasets presented in this study can be found in online repositories. The name of the repository and accession number can be found below: NCBI; PRJNA950454.

## Author contributions

Conceptualization: FQ-F. Formal analysis: FQ-F, AC-M, and JN-Z. Funding acquisition: FQ-F. Methodology: LG-P, RG-P, AC-M, JN-Z, EI-L, GC-R, and EG-R. Supervision: FQ-F, AC-M, and JN-Z. Writing—original draft: FQ-F, GC-R, and JN-Z. Writing—revision and editing: AC-M, EI-L, ER-M, AS-M, RS, GC-R, and EG-R. All authors contributed to the article and approved the submitted version.

## References

[B1] Agrawal P. K. Babu B. K. Saini N. (2015). “Omics of model plants,” in PlantOmics: the omics of plant science. Eds. Barh D. Khan M. S. Davies E. (New Delhi: Springer India), 1–32.

[B2] Apodaca-Sánchez M. A. Quintero-Benítez J. A. (2008). “Pudrición de mazorca,” in Manejo sustentable del maíz (Mexico: Universidad Auntónoma de Sinaloa, Fundación Produce Sinaloa, SAGARPA, Gobierno del estado de Sinaloa), 71–78.

[B3] Arora S. Steuernagel B. Gaurav K. Chandramohan S. Long Y. Matny O.. (2019). Resistance gene cloning from a wild crop relative by sequence capture and association genetics. Nat. Biotechnol. 37 (2), 139–143. doi: 10.1038/s41587-018-0007-9 30718880

[B4] Atanasova-Penichon V. Bernillon S. Marchegay G. Lornac A. Pinson-Gadais L. Ponts N.. (2014). Bioguided isolation, characterization, and biotransformation by fusarium verticillioides of maize kernel compounds that inhibit fumonisin production. Mol. Plant-Microbe Interact. 27 (10), 1148–1158. doi: 10.1094/Mpmi-04-14-0100-R 25014591

[B5] Baetsen-Young A. Chen H. Shiu S.-H. Day B. (2020). Contrasting transcriptional responses to *Fusarium virguliforme* colonization in symptomatic and asymptomatic hosts. Plant Cell 33 (2), 224–247. doi: 10.1093/plcell/koaa021 PMC8136916 33681966

[B6] Bashir M. R. Atiq M. Sajid M. Mohsan M. Abbas W. Alam M. W.. (2018). Antifungal exploitation of fungicides against *Fusarium oxysporum* f. sp. capsici causing fusarium wilt of chilli pepper in Pakistan. Environ. Sci. pollut. Res. 25 (7), 6797–6801. doi: 10.1007/s11356-017-1032-9 29264855

[B7] Bauters L. Stojilkovic B. Gheysen G. (2021). Pathogens pulling the strings: effectors manipulating salicylic acid and phenylpropanoid biosynthesis in plants. Mol. Plant Pathol. 22 (11), 1436–1448. doi: 10.1111/mpp.13123 34414650 PMC8518561

[B8] Bhuiyan N. H. Selvaraj G. Wei Y. D. King J. (2009). Gene expression profiling and silencing reveal that monolignol biosynthesis plays a critical role in penetration defence in wheat against powdery mildew invasion. J. Exp. Bot. 60 (2), 509–521. doi: 10.1093/jxb/ern290 19039100 PMC2651457

[B9] Bolger A. M. Lohse M. Usadel B. (2014). Trimmomatic: a flexible trimmer for illumina sequence data. Bioinformatics 30 (15), 2114–2120. doi: 10.1093/bioinformatics/btu170 24695404 PMC4103590

[B10] Bray N. L. Pimentel H. Melsted P. Pachter L. (2016). Near-optimal probabilistic RNA-seq quantification. Nat. Biotechnol. 34 (5), 525–527. doi: 10.1038/nbt.3519 27043002

[B11] Briones-Reyes D. Castillo-Gonz lez F. Ch vez-Servia J. L. Aguilar-Rincón V. Alba García-de C. d.L.. (2015). Respuesta de maíz nativo del altiplano mexicano a pudrición de mazorca, bajo infección natural. Agronomía Mesoamericana 26 (1), 73–85. doi: 10.15517/am.v26i1.16922

[B12] Dangond Araujo J. J. Guerrero Dallos J. A. (2006). Metodología para la determinación de residuos de fungicidas benzimidazólicos en fresa y lechuga por HPLC-DAD. Rev. Colombiana Química 35, 67–79.

[B13] de Sousa R. R. Osorio P. R. A. Nose N. P. E. de Arruda G. L. Ferreira T. P. D. Haesbaert F. M.. (2022). Detection and transmission of *Fusarium verticillioides* in corn seeds according to the plant stage. Acta Scientiarum-Agronomy 44, 1–11. doi: 10.4025/actasciagron.v44i1.53213

[B14] Esau K. (1965). Plant anatomy (New York: John Wiley & Sons).

[B15] Gai X. Dong H. Wang S. Liu B. Zhang Z. Li X.. (2018). Infection cycle of maize stalk rot and ear rot caused by *Fusarium verticillioides* . PloS One 13 (7), e0201588. doi: 10.1371/journal.pone.0201588 30063754 PMC6067754

[B16] García Pérez R. D. Velarde Félix S. Garzón Tiznado J. A. Ureta Tellez J. (2012). “Distribución geográfica y caracterización molecular de fusarium spp en el centro-sur de sinaloa y respuesta de variedades de maíz al ataque de este patógeno,” in Instituto nacional de investigaciones forestales, agrícolas y pecuarias. Ed. Culiacán C. (Mexico: Instituto Nacional de Investigaciones Forestales, Agrícolas y Pecuarias), 15–19.

[B17] González Huerta A. Vázquez García L. M. Sahagún Castellanos J. Rodríguez Pérez J. E. (2007). Rendimiento del maíz de temporal y su relación con la pudrición de mazorca. Agricultura técnica en México 33 (1), 33–42.

[B18] Görtz A. Oerke E. C. Steiner U. Waalwijk C. de Vries I. Dehne H. W. (2008). Biodiversity of *Fusarium* species causing ear rot of maize in Germany. Cereal Res. Commun. 36, 617–622. doi: 10.1556/CRC.36.2008.Suppl.B.51

[B19] Haile J. K. N’Diaye A. Sari E. Walkowiak S. Rutkoski J. E. Kutcher H. R.. (2020). Potential of genomic selection and integrating “Omics” Data for Disease Evaluation in Wheat. Crop Breeding Genet. Genomics 2 (4), e200016. doi: 10.20900/cbgg20200016

[B20] Hao W. Li A. P. Wang J. Y. An G. Guan S. (2022). Mycotoxin contamination of feeds and raw materials in China in year 2021. Front. Veterinary Sci. 9. doi: 10.3389/fvets.2022.929904 PMC9281542 35847652

[B21] Hussein H. M. Christensen M. J. Baxter M. (2003). Occurrence and distribution of *Fusarium* species in maize fields in new Zealand. Mycopathologia 156 (1), 25–30. doi: 10.1023/a:1021307023039 12715944

[B22] Kebede A. Z. Johnston A. Schneiderman D. Bosnich W. Harris L. J. (2018). Transcriptome profiling of two maize inbreds with distinct responses to gibberella ear rot disease to identify candidate resistance genes. BMC Genomics 19 (1), 131. doi: 10.1186/s12864-018-4513-4 29426290 PMC5807830

[B23] Kim S.-B. Van den Broeck L. Karre S. Choi H. Christensen S. A. Wang G.-F.. (2021). Analysis of the transcriptomic, metabolomic, and gene regulatory responses to puccinia sorghi in maize. Mol. Plant Pathol. 22 (4), 465–479. doi: 10.1111/mpp.13040 33641256 PMC7938627

[B24] Lambarey H. Moola N. Veenstra A. Murray S. Rafudeen M. S. (2020). Transcriptomic analysis of a susceptible African maize line to *Fusarium verticillioides* infection. Plants-Basel 9 (9), 1–23. doi: 10.3390/plants9091112 PMC7569872 32872156

[B25] Lanubile A. Ferrarini A. Maschietto V. Delledonne M. Marocco A. Bellin D. (2014). Functional genomic analysis of constitutive and inducible defense responses to *Fusarium verticillioides* infection in maize genotypes with contrasting ear rot resistance. BMC Genomics 15, 1–16. doi: 10.1186/1471-2164-15-710 25155950 PMC4153945

[B26] Lee M.-H. Jeon H. S. Kim S. H. Chung J. H. Roppolo D. Lee H.-J.. (2019). Lignin-based barrier restricts pathogens to the infection site and confers resistance in plants. EMBO J. 38 (23), e101948. doi: 10.15252/embj.2019101948 31559647 PMC6885736

[B27] Leslie J. F. Summerell B. A. (2007). The fusarium laboratory manual (USA, UK, Australia: Blackwell Publishing Ltd).

[B28] Leyva-Madrigal K. Y. Larralde-Corona C. P. Apodaca-Sanchez M. A. Quiroz-Figueroa F. R. Mexia-Bolanos P. A. Portillo-Valenzuela S.. (2015). *Fusarium* species from the *Fusarium fujikuroi* species complex involved in mixed infections of maize in northern sinaloa, Mexico. J. Phytopathol. 163 (6), 486–497. doi: 10.1111/jph.12346

[B29] Liu Y. Guo Y. Ma C. Zhang D. Wang C. Yang Q. (2016). Transcriptome analysis of maize resistance to *Fusarium graminearum* . BMC Genomics 17 (1), 477. doi: 10.1186/s12864-016-2780-5 27352627 PMC4924250

[B30] Lizárraga-Sánchez G. J. Leyva-Madrigal K. Y. Sánchez-Peña P. Quiroz-Figueroa F. R. Maldonado-Mendoza I. E. (2015). *Bacillus cereus* sensu lato strain B25 controls maize stalk and ear rot in sinaloa, Mexico. Field Crops Res. 176, 11–21. doi: 10.1016/j.fcr.2015.02.015

[B31] Love M. I. Huber W. Anders S. (2014). Moderated estimation of fold change and dispersion for RNA-seq data with DESeq2. Genome Biol. 15 (12), 1–21. doi: 10.1186/s13059-014-0550-8 PMC4302049 25516281

[B32] Marasas W. F. O. Riley R. T. Hendricks K. A. Stevens V. L. Sadler T. W. Gelineau-van Waes J.. (2004). Fumonisins disrupt sphingolipid metabolism, folate transport, and neural tube development in embryo culture and *in vivo*: a potential risk factor for human neural tube defects among populations consuming fumonisin-contaminated maize. J. Nutr. 134 (4), 711–716. doi: 10.1093/jn/134.4.711 15051815

[B33] Miedes E. Vanholme R. Boerjan W. Molina A. (2014). The role of the secondary cell wall in plant resistance to pathogens. Front. Plant Sci. 5 (358). doi: 10.3389/fpls.2014.00358 PMC4122179 25161657

[B34] Okello P. N. Petrović K. Kontz B. Mathew F. M. (2019). Eight species of *Fusarium* cause root rot of corn (Zea mays) in south Dakota. Plant Health Prog. 20 (1), 38–43. doi: 10.1094/php-11-18-0075-rs

[B35] Olivares-Garcia C. A. Mata-Rosas M. Pena-Montes C. Quiroz-Figueroa F. Segura-Cabrera A. Shannon L. M.. (2020). Phenylpropanoids are connected to cell wall fortification and stress tolerance in avocado somatic embryogenesis. Int. J. Mol. Sci. 21 (16), 1–23. doi: 10.3390/Ijms21165679 PMC7460882 32784357

[B36] Qiu J. B. Xu J. H. Dong F. Yin X. C. Shi J. R. (2015). Isolation and characterization of *Fusarium verticillioides* from maize in eastern China. Eur. J. Plant Pathol. 142 (4), 791–800. doi: 10.1007/s10658-015-0652-5

[B37] Riley R. T. Torres O. Matute J. Gregory S. G. Ashley-Koch A. E. Showker J. L.. (2015). Evidence for fumonisin inhibition of ceramide synthase in humans consuming maize-based foods and living in high exposure communities in Guatemala. Mol. Nutr. Food Res. 59 (11), 2209–2224. doi: 10.1002/mnfr.201500499 26264677 PMC4956729

[B38] Riley R. T. Voss K. A. Speer M. Stevens V. L. Waes J.-v. (2006). “Fumonisin inhibition of ceramide synthease:A possible risk factor for human nueral tube defects,” in Sphingolipid biology. Eds. Hirabayashi Y. Igarashi Y. Merrill. A. H. (Tokyo Berlin Heidelberg New York: Springer-Verlag).

[B39] Román S. G. (2017). Caracterización de genotipos de maíz (Zea mays l.) a la infección de fusarium verticillioides en diferentes fases del ciclo de vida de la planta y su correlación con marcadores moleculares de tipo SNPs (Instituto Politécnico Nacional: Matria en Recursos Naturales y Medio Ambiente).

[B40] Roman S. G. Quiroz-Chavez J. Villalobos M. Urias-Gutierrez V. Nava-Perez E. Ruiz-May E.. (2020). A global screening assay to select for maize phenotypes with a high tolerance or resistance to fusarium verticillioides (Sacc.) nirenberg rots. Agronomy-Basel 10 (12), 1–18. doi: 10.3390/agronomy10121990

[B41] Ross P. F. Nelson P. E. Richard J. L. Osweiler G. D. Rice L. G. Plattner R. D.. (1990). Production of fumonisins by *Fusarium moniliforme* and *Fusarium proliferatum* isolates associated with equine leukoencephalomalacia and a pulmonary edema syndrome in swine. Appl. Environ. Microbiol. 56 (10), 3225–3226. doi: 10.1128/aem.56.10.3225-3226.1990 2285324 PMC184928

[B42] Santiago R. Barros-Rios J. Malvar R. A. (2013). Impact of cell wall composition on maize resistance to pests and diseases. Int. J. Mol. Sci. 14 (4), 6960–6980. doi: 10.3390/ijms14046960 23535334 PMC3645672

[B43] Schrenk D. Bignami M. Bodin L. Chipman J. K. del Mazo J. Grasl-Kraupp B.. (2022). Assessment of information as regards the toxicity of fumonisins for pigs, poultry and horses. Efsa J. 20 (8), 1–26. doi: 10.2903/j.efsa.2022.7534 PMC9399829 36034321

[B44] Shin Y. Chane A. Jung M. Lee Y. (2021). Recent advances in understanding the roles of pectin as an active participant in plant signaling networks. Plants-Basel 10 (8), 1–22. doi: 10.3390/plants10081712 PMC8399534 34451757

[B45] SIAP (2021) Servicio de información agroalimentaria y pesquera. anuario estadístico de la producción agrícola. Available at: https://nube.siap.gob.mx/cierreagricola/ (Accessed April/27/2023).

[B46] Steuernagel B. Periyannan S. K. Hernández-Pinzón I. Witek K. Rouse M. N. Yu G.. (2016). Rapid cloning of disease-resistance genes in plants using mutagenesis and sequence capture. Nat. Biotechnol. 34 (6), 652–655. doi: 10.1038/nbt.3543 27111722

[B47] Supek F. Bosnjak M. Skunca N. Smuc T. (2011). REVIGO summarizes and visualizes long lists of gene ontology terms. PloS One 6 (7), 1–9. doi: 10.1371/journal.pone.0021800 PMC3138752 21789182

[B48] Vanholme R. De Meester B. Ralph J. Boerjan W. (2019). Lignin biosynthesis and its integration into metabolism. Curr. Opin. Biotechnol. 56, 230–239. doi: 10.1016/j.copbio.2019.02.018 30913460

[B49] Wan J. He M. Hou Q. Zou L. Yang Y. Wei Y.. (2021). Cell wall associated immunity in plants. Stress Biol. 1 (1), 3. doi: 10.1007/s44154-021-00003-4 PMC10429498 37676546

[B50] Wang Z. P. Li Y. J. Li C. N. Song X. P. Lei J. C. Gao Y. J.. (2019). Comparative transcriptome profiling of resistant and susceptible sugarcane genotypes in response to the airborne pathogen *Fusarium verticillioides* . Mol. Biol. Rep. 46 (4), 3777–3789. doi: 10.1007/s11033-019-04820-9 31006101

[B51] Wang Y. P. Li T. Sun Z. D. Huang X. J. Yu N. B. Tai H. H.. (2022). Comparative transcriptome meta-analysis reveals a set of genes involved in the responses to multiple pathogens in maize. Front. Plant Sci. 13. doi: 10.3389/fpls.2022.971371 PMC9521429 36186003

[B52] Wang B. B. Lin Z. C. Li X. Zhao Y. P. Zhao B. B. Wu G. X.. (2020). Genome-wide selection and genetic improvement during modern maize breeding. Nat. Genet. 52 (6), 565–56+. doi: 10.1038/s41588-020-0616-3 32341525

[B53] Warham E. J. Butler L. D. Sutton B. C. (1996). Seed testing of maize and wheat: a laboratory guide (Ciudad de México: Cimmyt).

[B54] Wu T. Z. Hu E. Q. Xu S. B. Chen M. J. Guo P. F. Dai Z. H.. (2021). ClusterProfiler 4.0: a universal enrichment tool for interpreting omics data. Innovation 2 (3), 1–11. doi: 10.1016/j.xinn.2021.100141 PMC8454663 34557778

[B55] Wu L. Wang X.-M. Xu R.-Q. Li H.-J. (2011). Root infection and systematic colonization of DsRed-labeled *Fusarium verticillioides* in maize. Acta Agronomica Sin. 37 (5), 793–802. doi: 10.1016/S1875-2780(11)60023-0

[B56] Yadav S. Chattopadhyay D. (2023). Lignin: the building block of defense responses to stress in plants. J. Plant Growth Regulation. doi: 10.1007/s00344-023-10926-z

[B57] Yadav V. Wang Z. Y. Wei C. H. Amo A. Ahmed B. Yang X. Z.. (2020). Phenylpropanoid pathway engineering: an emerging approach towards plant defense. Pathogens 9 (4). doi: 10.3390/pathogens9040312 PMC7238016 32340374

[B58] Yu Y. Shi J. Li X. Liu J. Geng Q. Shi H.. (2018). Transcriptome analysis reveals the molecular mechanisms of the defense response to gray leaf spot disease in maize. BMC Genomics 19 (1), 742. doi: 10.1186/s12864-018-5072-4 30305015 PMC6180411

[B59] Zhou Z. J. Cao Y. Li T. Wang X. H. Chen J. F. He H.. (2020). MicroRNAs are involved in maize immunity against *Fusarium verticillioides* ear rot. Genomics Proteomics Bioinf. 18 (3), 241–255. doi: 10.1016/j.gpb.2019.11.006 PMC7801212 32531477

[B60] Zhu Y. Abdelraheem A. Cooke P. Wheeler T. Dever J. K. Wedegaertner T.. (2022). Comparative analysis of infection process in pima cotton differing in resistance to *Fusarium* wilt caused by *Fusarium oxysporum* f. sp. vasinfectum race 4. Phytopathology 112 (4), 852–861. doi: 10.1094/Phyto-05-21-0203-R 34503350

