# Supplementary material for: Cell wall-related genes and lignin accumulation contribute to the root resistance in different maize (Zea mays L.) genotypes to Fusarium verticillioides (Sacc.) Nirenberg infection
Source: Front Plant Sci. 2023 Jun 27;14:1195794. doi: 10.3389/fpls.2023.1195794 (PMC10335812; doi:10.3389/fpls.2023.1195794)
Supplement: Supplementary file 1 [file DataSheet_1.docx]

Supplementary Material

Differences in cell wall organization and gene expression are essential in the resistance of different maize (Zea mays L.) genotypes to Fusarium verticillioides (Sacc.) Nirenberg infection

Francisco Roberto Quiroz-Figueroa^1,*,†^, Abraham Cruz-Mendívil^1,†^, Enrique Ibarra-Laclette^3^, Luz María García-Pérez^1^, Rosa Luz Gómez-Peraza^1^, Greta Hanako-Rosas^2^, Eliel Ruíz-May^2^, Apolinar Santamaría-Miranda^1^, Rupesh Kumar Singh^3^, Gerardo Campos-Rivero^1^, Elpidio García-Ramírez^4^, José Alberto Narváez-Zapata^5^

*** Correspondence:** Corresponding Author: [fquiroz@ipn.mx](mailto:fquiroz@ipn.mx) or [labfitomol@hotmail.com](mailto:labfitomol@hotmail.com)


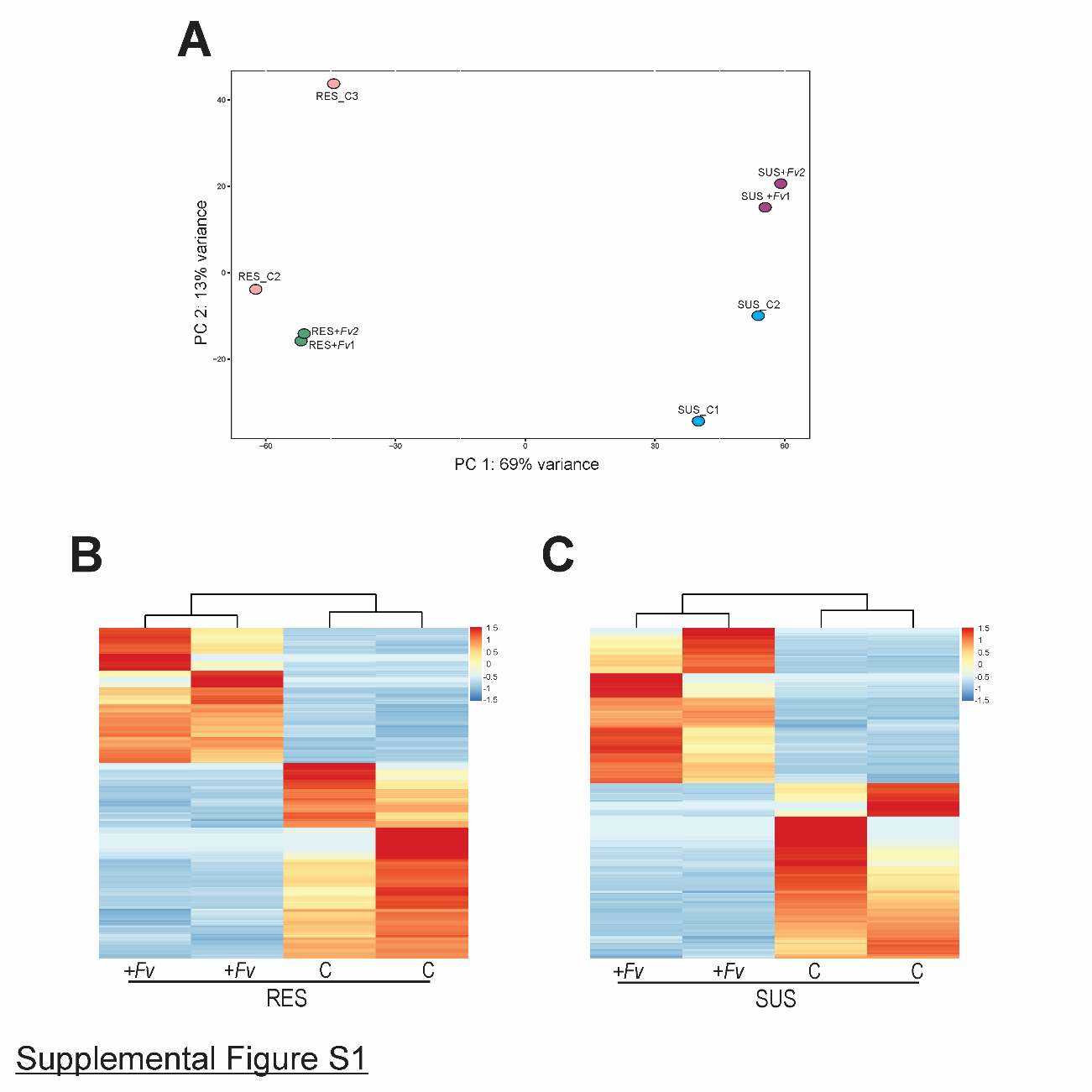


**Supplementary Figure S1.** Reproducibility and biological variation among samples were verified by **A**) principal component analysis (PCA) and heatmaps of **B**) resistant (RES) and **C**) susceptible (SUS) genotypes that were either uninfected (C) or infected with *F. verticillioides* (+*Fv*).


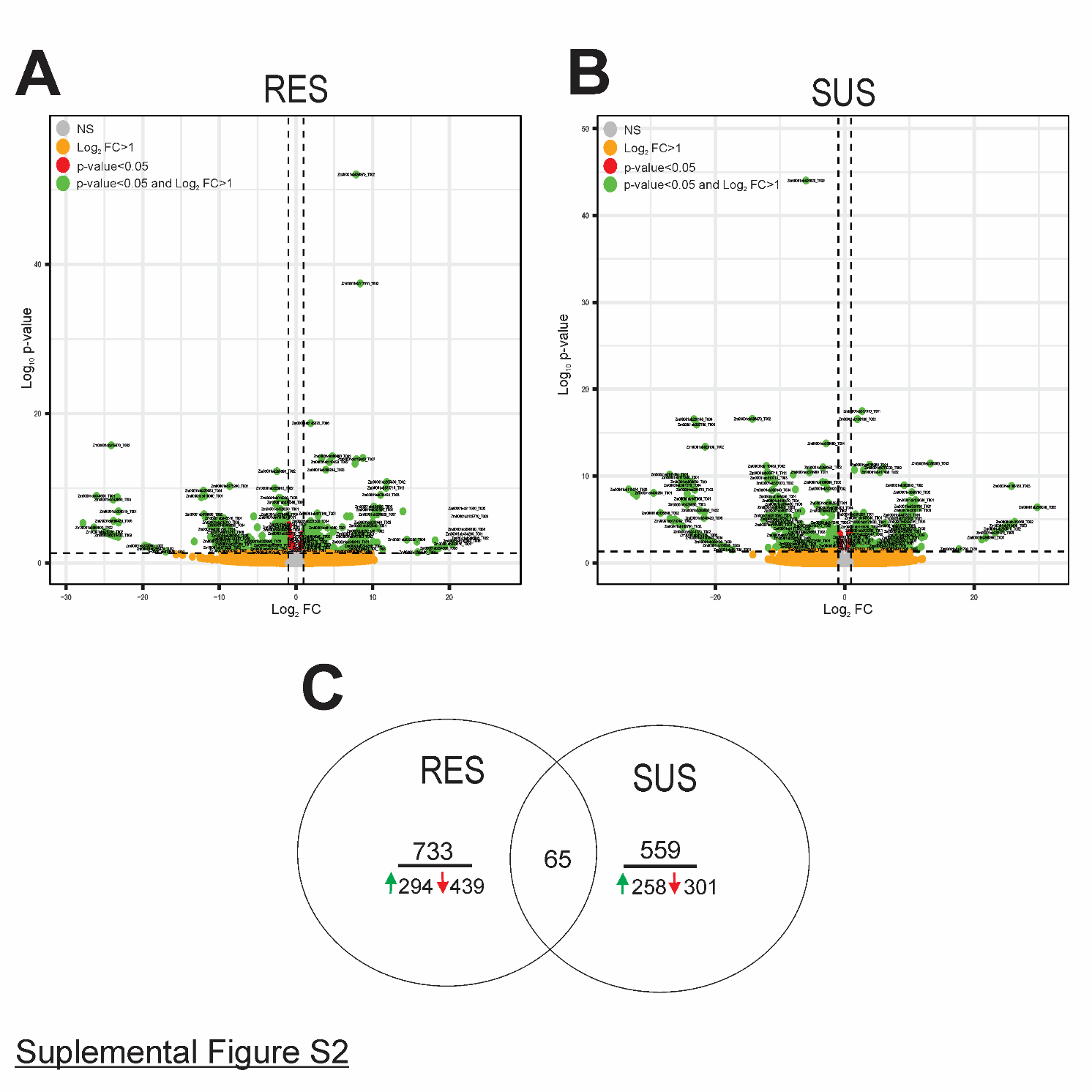


**Supplementary Figure S2.** Volcano plots show the gene expression rate of infected/uninfected (+*Fv*/C) maize RNA-Seq data for **A**) RES genotype and **B**) SUS genotype. Differentially expressed genes (DEGs, green circles) were defined with adjusted P-value (False Discovery Rate, FDR) < 0.05 and by log2 fold change > 1.0. The x-axis represents the log fold change of gene expression and the y-axis represents the statistical significance (-log10 of the p-value). **C**) Venn diagram of the DEG overlap between RES and SUS genotypes.

**Supplementary Table 1.** Summary of trimming and alignment of RNA-Seq reads.

**Supplementary Table 4.** KEGG IDs involved in the enrichment analysis.
