# Supplementary figures and images for: Cell wall-related genes and lignin accumulation contribute to the root resistance in different maize (Zea mays L.) genotypes to Fusarium verticillioides (Sacc.) Nirenberg infection
Source: Front Plant Sci. 2023 Jun 27;14:1195794. doi: 10.3389/fpls.2023.1195794 (PMC10335812; doi:10.3389/fpls.2023.1195794)

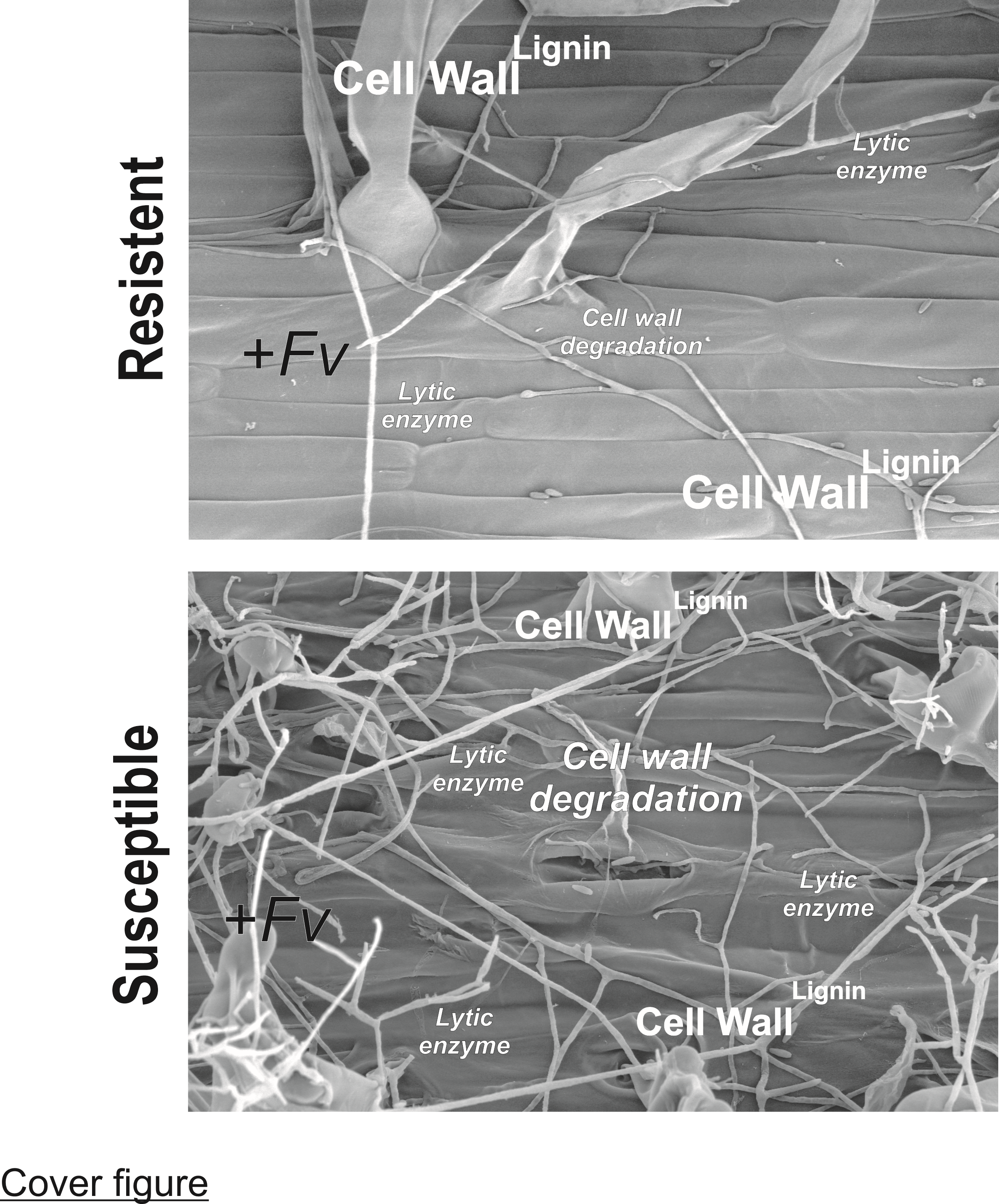

Supplement: Supplementary file 2 [file Image_1.tif]
